# A Retrospective Study to Examine the Correlation of Bioelectrical Impedance Analysis with Shear-wave Elastography in Indian Patients with Non-alcoholic Fatty Liver Disease and Diabetes on Background Sodium-glucose Cotransporter-2 Inhibitor Therapy

**DOI:** 10.7759/cureus.4674

**Published:** 2019-05-15

**Authors:** Sayak Roy, Anirban Majumder

**Affiliations:** 1 Internal Medicine, Calcutta Medical Research Institute Hospital, Kolkata, IND; 2 Endocrinology, Kali Prasad Chowdhury Medical College & Hospital, Kolkata, IND

**Keywords:** shearwave elastography, bioimpedance analysis, sdodium glucose co-transporter 2 inhibitor, type 2 diabetes mellitus, non-alcoholic fatty liver diseaseintroduction

## Abstract

Background

Patients with non-alcoholic fatty liver disease (NAFLD) are often insulin resistant. Several recent studies show NAFLD to be associated with cardiovascular risk. Bioimpedance analysis (BIA) is a common approach for body composition measurements and is a noninvasive, low-cost modality. Shear-wave elastography (SWE) works using an acoustic radiation force pulse sequence that generates shear-waves that estimates the liver stiffness.

Objectives

The primary objective was to assess the correlation between SWE values and BIA values in an Indian population. The hypothesis is that with the increase in BIA value measuring visceral fat percentage, the SWE value measuring liver stiffness should increase.

Materials and methods

We conducted a retrospective analysis of clinic data of 36 patients properly screened from July 2018 to December 2018, who matched our prespecified inclusion criteria. Statistical analysis was performed using GraphPad Insta Version 3.0^®^ using regression analysis. Visceral fat percentage and skeletal muscle percentage of lower limbs were calculated using an Omron HBF 375^®^ analyzer. SWE values for liver fat were measured using a Philips Affinity 70^®^ using two-dimensional imaging and expressed in kilopascal (kPa) units.

Results

We found that 88.88% of the patients with diabetes had above normal SWE values (2.0 to 4.5 kPa), and a corresponding 83.33% of patients had above the high cut-off for BIA values (>10%) but without any positive correlation between the two parameters as evident from the *p*-value of 0.079.

Conclusions

This study found a high prevalence of fat burden amongst our patients with type 2 diabetes and NAFLD. This is the first of its kind of study where we searched for a correlation between the two commonly used parameters in assessing the fat burden and liver stiffness of an individual but found there was no significant correlation between the two parameters used.

## Introduction

Non-alcoholic fatty liver disease (NAFLD), diabetes, and cardiovascular disease (CVD) all share a common origin. On ultrasound (US), hepatic steatosis appears as diffusely increased hepatic echogenicity and is often called “bright liver”. This is due to increased reflection of the US waves from the bed of the liver parenchyma, caused due to fat vacuole accumulation in the intracellular space. The evaluation process of hepatic steatosis by US depends on the qualitative visual assessment of echogenicity of liver parenchyma that is then used to determine differences in measurements between the echo-amplitude of the liver and kidneys [1]. Traditionally, liver biopsy is regarded as the gold standard in the diagnosis of NAFLD. However, due to its invasiveness, expense, and the possibility of complications, its use is now limited [2]. Hence, it seems essential to reappraise and reassess the diagnostic ability of other emerging noninvasive modalities. Recently, US elastography has been developed as a noninvasive modality for the evaluation of liver fibrosis, and it aims to replace the more invasive and risky liver biopsy procedure. US elastography is affected by the degree of liver fibrosis present in most chronic hepatic disease states; hence, it can be used for measuring the mechanical properties of liver tissues. An ultrasonic transducer is used to perform elastography in combination with any of the three shear-wave (SW) techniques available, such as transient elastography, point SW elastography (SWE), and two-dimensional SWE [3]. Shear waves produced by a focused US beam are found to be directly related to the liver stiffness; this is the basic principle of interpretation of SWE [4-5]. Repeat measurements can be taken in patients with progressive chronic liver diseases due to its noninvasive nature. However, this method has some pitfalls: there is intra- and inter-observer variability; readings in individuals with hepatitis C have the only validated cut-offs; false positive results are associated with acute hepatitis. Other problems that may arise in patients with a high body mass index (BMI), where it can give erroneous values. Other confounding factors are cholestasis, congestion, edema, and inflammation [6-7]. The SWE method uses the values of acoustically-produced SW propagation speeds in liver tissue then estimates the liver stiffness with the bonus advantage of simultaneous real-time anatomic B-Mode US imaging [6]. Generally, the stiffer a tissue is, the greater its SW value will be as the wave travels through this region. The measured SW value thus becomes an intrinsic and reproducible property of the tissue. The use of the sodium-glucose cotransporter-2 (SGLT2) inhibitor (SGLT2i) canagliflozin at 300 mg has been shown to correlate with a reduction in SWE values to a significant extent even in a population without diabetes [8]. SGLT2is allow urinary glucose excretion that, in turn, leads to calorie loss and weight loss along with osmotic diuresis. This calorie loss and decrease in insulin resistance lead to visceral fat loss from the liver. This property has been tested in many studies, and all proved to be quite fruitful [9-11]. Hepatic steatosis improvement by SGLT2is has been linked to the inhibition of serum soluble dipeptidyl peptidase 4 (DPP4) enzyme secreted by hepatocytes and insulin resistance [12].

Being noninvasive and due to their easy availability, using bioimpedance analysis (BIA) machines in clinics is becoming a common practice to get an overview of the visceral fat mass of the patient. Many studies have incorporated BIA parameters in their randomized studies to assess changes in visceral fat scores with drug interventions. We conducted this study to assess the correlation between SWE values and BIA values in an Indian population.

## Materials and methods

Materials

We conducted a retrospective review of clinic records of 36 patients (22 men and 14 women) seen between July 2018 to December 2018 matching the pre-specified inclusion criteria: type 2 diabetes with sonographically established fatty liver disease with the fat burden quantified by SWE; data of BIA for visceral fat and skeletal muscle lower limb values measured within one week around the time of measurement of SWE; elevated serum glutamic pyruvic transaminase and serum glutamic oxaloacetic transaminase blood levels >1.5 times the upper limit of normal; negative for hepatitis B and C; abstaining from alcohol; glycosylated hemoglobin (HbA1c) of 6.5% to 12%; non-pregnant, non-type 1 diabetes mellitus (T1DM), and non-latent autoimmune diabetes in adults; a duration of diabetes at least six months; BMI <30; age >19 years; estimated glomerular filtration rate (eGFR) as per chronic kidney disease epidemiology collaboration equation ≥ 45 ml/min; treatment of any chronic disease must have been on standard treatment for more than six months; no approved anti-obesity drug intake in last six months; no known chronic liver disease as per history and relevant questions; stable treatment with any oral hypoglycemic drugs (except pioglitazone and saroglitazar) for six months, and no electronic implant devices such as pacemaker or implantable cardioverter defibrillator.

Written informed consent was obtained from all patients. All of them had type 2 diabetes and NAFLD as per the history provided and the sonological report. They were having SWE performed and reported as well as BIA values, with both being done within one week. This study was executed as a retrospective data collection. The various forms of SGLT2i used were dapagliflozin 5 mg (n=1) and 10 mg (*n *= 13), empagliflozin 12.5 mg (*n *= 3) and 25 mg (*n *= 7), and canagliflozin 100 mg (*n *= 7) and 300 mg (*n *= 5). Statins (atorvastatin 10 mg, or rosuvastatin 10 mg) were used in those patients having dyslipidemia as defined by either low-density lipoprotein >100 mg/dL or non-high-density lipoprotein > 130 mg/dL or having cardiovascular (CV) risk factors. Either teneligliptin 20 mg or glimepiride was used at various doses. The common drugs used in all the patients were different classes of SGLT2is and metformin at various doses. Baseline drugs used other than SGLT2is are depicted in Table 1.

**Table 1:** Baseline drugs used in the population and their percentages (other than SGLT2is) SGLT2i, sodium-glucose cotransporter 2 inhibitor

Drug names with dose (mg)	Percentage of users (%)	Number of users (n)
Glimepiride 1	11.11%	4
Glimepiride 2	11.11%	4
Glimepiride 3	2.77%	1
Glimepiride 4	25%	9
Metformin 500	25%	9
Metformin 1000	36.11%	13
Metformin 1500	19.44%	7
Metformin 2000	19.44%	7
Teneligliptin 20	50%	18
Atorvastatin 10	27.77%	10
Rosuvastatin 10	16.66%	6

Objectives of the study

The primary objective of the study was to evaluate the correlation between SWE values and BIA values in the study population. The secondary objectives of the study were to determine the burden of increased SWE scores above normal, and BIA score above 10 in the population; examine the correlation between different SWE values within different HbA1c categories; evaluate the correlation between different eGFR values with different SWE values; evaluate the correlation between different SGLT2i class and BIA, SWE, HbA1c, skeletal muscle percentage of the lower limb (SKM.L/LIMB%), and eGFR values; evaluate the correlation between SWE and SKM.L/LIMB% (women/men) BIA values; evaluate the correlation between SWE and BMI; and to perform and evaluate a subgroup analysis between two cohorts: one using sulfonylurea + metformin + SGLT2i and the other using teneligliptin + metformin + SGLT2i to look for any difference between the groups in terms of age, HbA1c, eGFR, BIA visceral fat percentage of the abdomen (VIS.FAT%), BIA skeletal muscle percentage of the lower limb (SKM.L/LIMB%), SWE value, and BMI.

Methods

BIA was performed using an Omron HBF 375® to assess visceral fat score percentages and skeletal muscle lower limb percentages. SWE using Philips Affinity 70® was used to assess liver stiffness. The Omron HBF 375 estimates the fat percentage of the body by sending an extremely weak and negligible electrical current of 50 kHz and always less than 500 μA through the body to estimate the percentage of water in each tissue via the bioelectrical impedance (BI) method. Blood, muscles, body tissues, and bones have high water content, and so they conduct electricity easily.

On the contrary, our body fat contains much less water and thus conducts little electric impulse. The definitions of the cut-off for increased visceral fat percentage and the skeletal muscle lower limb percentage were taken from the product monograph-provided reference values (Table 2).

**Table 2:** Pre-specified reference values for skeletal muscle percentage of the lower limb and visceral fat percentage from the Omron HBF 375® product monograph

Visceral fat percentage values	Skeletal muscle percentage of the lower limb for men	Skeletal muscle percentage of the lower limb for women
Normal = 0.5–9.5	Low = 5.0–32.8	Low = 5.0–25.8
High = 10–14.5	Normal = 32.9–35.7	Normal = 25.9–27.9
Very high = 15–30	High = 35.8–37.3	High = 28–29
	Very high = 37.4–60	Very high = 29.1–60

BIA uses the electrical properties of the body to estimate the total body water. This value is then applied to assess the body fat mass as well as the body visceral fat percentage [13]. SWE using a Philips Affinity 70® was performed to assess visceral fat scores as measured in kilopascals (kPa). The underlying principle behind the interpretation of SWE is that the SWs produced by a focused US beam are found to be directly related to the stiffness of the liver tissue area through which the wave propagates and hence it an intrinsic property of the tissue itself [14]. The SWE value is generally expressed in kPa when we are using the METAVIR scoring system or m/sec when we are using acoustic radiation frequency impulse (ARFI) system. There are generally four stages depending on the METAVIR score or ARFI grades (Table 3).

**Table 3:** METAVIR score and classification of liver stiffness depending on SWE values and corresponding acoustic radiation force impulse grades with values ARFI, acoustic radiation force impulse; SWE, shear-wave elastography

Classification	METAVIR scores	Range (kPa)	ARFI grade	ARFI values (m/sec)
Normal	F0	2.0–4.5	Normal	1.0–1.5
Normal to mild	F0–F1	4.6–5.7	Mild fibrosis	1.5–1.75
Mild to moderate	F2–F3	5.8–12	Moderate fibrosis	1.75–2.1
Moderate to severe	F3–F4	12.1–21.0+	Severe fibrosis	>2.1

Baseline data for individual HbA1c, eGFR, BMI, duration of diabetes, age, duration of dyslipidemia, and detailed drug history were also taken to get an overall view of the retrospective data.

Statistical analysis

Data were analyzed using GraphPad Insta Version 3.0®. A regression analysis was done to calculate the correlation of the various individual parameters to each other, and a multilinear regression analysis was done to see the correlation of the cross-sectional variables with respect to each class of SGLT2i used. A subgroup analysis was done dividing this population into two different cohorts. The first cohort consisted of 18 patients on glimepiride + metformin + SGLT2i and the second cohort consisted of the remaining 18 patients on teneligliptin + metformin + SGLT2i. *P-*value was calculated using the Mann-Whitney U test (a nonparametric test). The individual component of this subgroup analysis has been represented using a box and whisker plot. A *P-* value of <0.05 would be considered statistically significant.

## Results

The study population had a median entry BMI of 27.75, a median age of 52 years, and a median duration of diabetes of six years. The median HbA1c of the population was 7.25%, the median visceral fat score was 12%, and the median SWE value was 6.72 kPa (1.5 m/sec). A regression analysis done on various baseline parameters taken over a cross-sectional period, plotted in Table 4, showed no positive correlation of any baseline parameters with each other except for a positive correlation between the baseline duration of diabetes and SWE values; this was statistically significant (*P* = 0.049).

**Table 4:** Regression analysis of various baseline parameters with each other BIA, bioelectrical impedance analysis; SWE, shear-wave elastography; HbA1c, glycosylated hemoglobin; BMI, body mass index, eGFR; estimated glomerular filtration rate

Correlation done between variables	R^2^ Value	P value	Interpretation
SWE vs BIA visceral fat percentage	0.088	0.079	Nonsignificant
HbA1c vs SWE	0.001764	0.808	Nonsignificant
HbA1c vs Visceral fat percentage (BIA)	0.039	0.243	Nonsignificant
HbA1c vs Skeletal muscle percentage of the lower limb in men	0.08040	0.170	Nonsignificant
HbA1c vs Skeletal muscle percentage of the lower limb in women	0.3012	0.080	Nonsignificant
eGFR vs SWE values	0.0045	0.698	Nonsignificant
SWE vs BMI	0.074	0.107	Nonsignificant
SWE vs Duration of diabetes	0.1094	0.049^*^	Statistically significant

A multiple regression analysis was done to assess any correlation between baseline parameters with all the classes of SGLT2i used in the study. It was observed that the group taking canagliflozin had a significant correlation of their HbA1c values to the BIA visceral fat percentage (*P* = 0.0047). However, when a paired t-test was applied to this result, the correlation becomes nonsignificant (*P* = 0.9086), meaning that due to the small sample size, we cannot properly conclude this fact. Similarly, there was a significant correlation in the empagliflozin group between baseline eGFR values and HbA1c values in multiple regression analysis (*P* = 0.0330), but on applying a paired t-test to this data, the result was nonsignificant (*P* = 0.5923) making any conclusion drawn on this data impossible due to small sample size. None of the parameters had any significant correlation to the dapagliflozin group in the multiple linear regression analysis.

On analyzing the SWE reports, we noted most of the patient results (52.77 %) were in the F2 to F3 METAVIR score range for liver stiffness corresponding to a mild-to-moderate stage of liver stiffness. Table 5 shows the distributions of the patients as per their various METAVIR scores. After correlating BIA with METAVIR scores, the average BIA for visceral fat percentage is in the order of F3 to F4 > F0 > F2 to F3 > F0 to F1. The average BIA values do not correspond to the METAVIR stage as can be easily seen in Table 5.

**Table 5:** Population distribution and percentage as per various METAVIR scores and their corresponding average BIA (visceral fat) percentage BIA, bioelectrical impedance analysis

METAVIR score	Number of patients (n)	Percentage in total population (%)	Average BIA percentage in a METAVIR stage (%)
F0	4	11.11%	13.125%
F0 – F1	10	27.77%	9.504%
F2 – F3	19	52.77%	12.105%
F3 – F4	3	8.33%	15.0%

Subgroup analysis between two cohorts

A subgroup analysis was performed on this population by dividing them into two different cohorts. The first cohort consisted of 18 patients on a glimepiride + metformin + SGLT2i combination and the second cohort consisted of the remaining 18 patients on teneligliptin + metformin + SGLT2i. The analysis was done to see any baseline differences between the cross-sectional parameters used for the study between the two cohorts. The analysis explored the difference in terms of age, HbA1c, BMI, visceral fat percentage, skeletal muscle percentage of the lower limb, eGFR, and SWE value as measured in kPa (Table 6).

**Table 6:** Comparative analysis between two cohorts (glimepiride + metformin + SGLT2i versus teneligliptin + metformin + SGLT2i) in terms of age, BMI, HbA1c, VIS.FAT%, SKM L.LIMB%, eGFR, and SWE (kPa) BIA, bioelectrical impedance analysis; SWE, shear-wave elastography; HbA1c, glycosylated hemoglobin; BMI, body mass index, eGFR; estimated glomerular filtration rate; SGLT2i, sodium-glucose cotransporter 2 inhibitor; VIS.FAT%, visceral fat; SKM L.LIMB%, skeletal muscle percentage of the lower limb; Met, meformin; SU, sulfonylurea; Teneli, teneligliptin

Cohort studied			Age	BMI	HbA1c	VIS. FAT %	SKM. L.LIMB	EGFR	SWE (kPa)
SU+Met+SGLT2i	Mean		52.67	27.41	7.89	11.81	38.96	88.06	6.84
	Median		52.00	27.70	7.20	11.50	37.15	94.00	5.50
	Percentiles	Lower Quartile	47.75	26.08	6.88	9.75	34.50	70.50	4.87
		Upper Quartile	60.50	29.03	8.85	14.25	44.28	105.25	8.25
Teneli+Met+SGLT2i	Mean		50.94	27.86	7.42	12.33	41.32	87.50	8.33
	Median		51.00	27.75	7.25	12.00	42.50	85.50	7.00
	Percentiles	Lower Quartile	45.50	26.98	6.68	10.75	38.63	75.25	6.00
		Upper Quartile	59.50	29.63	7.65	14.00	44.98	101.75	10.25
P value (Mann-Whitney U test)			0.606	0.628	0.696	0.501	0.323	0.815	0.059

Individual analysis revealed that any difference between the two cohorts in terms of BMI, age, HbA1c, visceral fat percentage, skeletal muscle percentage of the lower limb, estimated glomerular filtration rate, and SWE value were all statistically nonsignificant after determining the P value using a Mann-Whitney U test for each parameter. This confirmed that the baseline drug use for any duration did not impact the parameters assessed and our hypothesis that with an increase in BIA visceral fat percentage there should be an increase in SWE values was proven wrong as these parameters did not correlate with each other.

## Discussion

Due to the noninvasive property, low cost, and portable, easy-to-carry size of BIA systems, many researchers have conducted studies using BIA methods with these machines. They have found applications in the evaluation of clinical conditions and body composition estimation. BIA has also been used to detect obesity in an Iranian study that also showed a significant correlation (*P* < .001) between body fat percentage and BMI of men and women [15]. BIA obtained at 50 KHz electric current is also known as single-frequency BIA (SF-BIA). SF-BIA has become the most common and one of the earliest methods for estimating body compartments. BIA obtained from more than two frequencies is known as multiple-frequency BIA (MF-BIA). MF-BIA uses the principle that the extra-cellular fluid and total body weight assay can be done by exposing it to both low- and high-frequency electric currents, respectively [16]. A study using the BIA Inbody 770® showed a significant reduction in fat percentage in patients with diabetes in the SGLT2i group having pretreatment HbA1c levels of more than 7.7% [17]. In another study, tofogliflozin was used in 17 patients with diabetes over only eight weeks, and body composition changes were analyzed using an Inbody-S20®. This study saw a significant decrease in HbA1c levels, free fat mass, total body water, extracellular water, and intracellular water; these all decreased significantly [18]. Another study, conducted to validate the results of BIA with the visceral fat area as measured by computed tomography (CT) scan in 103 patients, showed the method of estimating the visceral fat area using multifrequency BI is simple and convenient and can be easily used to estimate accurately visceral fat area [19].

SGLT2is, by their action of urinary glucose excretion, cause a decrease in insulin resistance and loss of visceral fat [20]. In a Japanese single-center, open-label study involving five patients, the effect of canagliflozin of 100 mg was observed on serial liver biopsy specimens at baseline and after 24 weeks of treatment to have significantly reduced NAFLD score as well as rates of hepatocyte steatosis [21]. There was also significant reduction in BMI (*P *= 0.042), waist circumference (*P* = 0.043), gamma-glutamyl transpeptidase (*P* = 0.042), fasting plasma glucose (*P* = 0.043), type IV collagen 7S (*P* = 0.043), serum ferritin (*P* = 0.043), and liver stiffness measurement (*P* = 0.043). In another retrospective analysis of 33 patients, the use of SGLT2is for 12 weeks caused a significant reduction in mean HbA1c (*P* = 0.014), visceral fat as measured by Omron HBF 375 BIA analyzer® (*P* = 0.0027), and waist circumference (*P* = 0.012) [22]. After applying multiple regression analysis on the data, the change in HbA1c was affected by baseline BMI, skeletal muscle trunk percentage, visceral fat percentage, and baseline HbA1c.

To the best of our knowledge, this is the first study of its kind that has tried to correlate two commonly used methods for fat assessment and liver stiffness measurement as a surrogate for each other. The study failed to show any such correlation, however. Though this is a small study with a few setbacks in terms of entry criteria used, it still can provide some direction in looking for the correlation in a larger population with well-defined inclusion criteria. As SWE and BIA machines are easily available and can be used without any intervention on the patients and repeated innumerable times, a database of results should be generated for each test that can then be used to correlate both results to look for any possible positive associations between the two for different ethnicities, BMI, HbA1c, and other factors so that clinicians may easily judge the CV risks for patients with diabetes and NAFLD. The study shows the high percentage of the existing visceral fat load as assessed by BIA score of above 10% in 80.55% (*n* = 29) of patients and liver stiffness values of >5.7 kPa as measured by SWE in 66.66% (*n *= 24). South Asians are already at risk of increased hypertension, visceral adiposity, metabolic syndrome, type-2 diabetes, and vascular diseases [23] and easy methods to calculate visceral adiposity that correlates with CVD risk are needed. For this, BIA assessment or SWE assessment can be of great help.

There were some limitations to our study. First, magnetic resonance (MR) spectroscopy, the gold standard, was not used for visceral fat assessment. Also, this study had a small sample size. There was no specified upper limit of entry criteria with respect to duration of diabetes. This study did not capture the initiation time of SGLT2i use at entry level. This study did not obtain information on the previous drugs used prior to on-going drugs. No information was obtained concerning any lifestyle changes made by the patient. Lastly, the usual class of evidence for any cohort study is II or III. Any other bias associated with any retrospective study is also applicable to this one.

## Conclusions

NAFLD, diabetes, and CVD all share a common ground in inflammation. This study shows a high prevalence of fat burden amongst our patients with type 2 diabetes and NAFLD as assessed by SWE values. This speaks to the higher prevalence of CVD in India. This is the first-of-its-kind study where we searched for a correlation between the two commonly used parameters in assessing fat burden and liver stiffness for an individual but found no significant correlation between the two parameters used. We need larger studies with a much larger number of patients to make a database of these two common methods for visceral fat assessment depending on the ethnicity, duration of diabetes, baseline BMI, baseline HbA1c, and other CV risk factors and then correlate the value of each method with the particular value of MR spectroscopic fat score for the benefit of clinicians at all levels.
